# Effect of HLA restriction on racial and ethnic disparities in access to immune therapies for advanced synovial sarcoma

**DOI:** 10.1093/oncolo/oyaf193

**Published:** 2025-07-16

**Authors:** Vinayak Venkataraman, Hannah R Abrams, David S Shulman, Elizabeth T Loggers, Seth M Pollack, Kelly G Paulson, Michael J Wagner

**Affiliations:** Department of Medical Oncology, Dana-Farber Cancer Institute, Boston, MA 02215, United States; Department of Medicine, Harvard Medical School, Boston, MA 02115, United States; Clinical Research Division, Fred Hutch Cancer Center, Seattle, WA 98109, United States; Department of Medicine, Harvard Medical School, Boston, MA 02115, United States; Solid Tumor Center, Dana-Farber/Boston Children’s Cancer and Blood Disorders Center, Boston, MA 02215, United States; Clinical Research Division, Fred Hutch Cancer Center, Seattle, WA 98109, United States; Department of Medicine, Northwestern University, Chicago, IL 60611, United States; Department of Medical Oncology, Providence-Swedish Cancer Institute, Seattle, WA 98026, United States; Department of Medical Oncology, Dana-Farber Cancer Institute, Boston, MA 02215, United States; Department of Medicine, Harvard Medical School, Boston, MA 02115, United States

**Keywords:** synovial sarcoma, cell therapy, immunology, disparities

## Abstract

**Purpose:**

Synovial sarcoma (SS) is aggressive with poor outcomes. Cellular therapies are now FDA-approved for advanced disease, but are restricted to certain HLA-A*02 alleles. We estimate eligibility for cellular therapies by race and ethnicity.

**Materials and Methods:**

Demographic and clinical features of SS cases from 2001 to 2020 were obtained from the United States Cancer Statistics (USCS; NPCR-SEER). Survival analyses were performed overall and by race/ethnicity. The proportion eligible for cellular therapy was estimated by race/ethnicity using previously published data on HLA-A*02 status and MAGE-A4 positivity.

**Results:**

From 2001 to 2020, 10 605 patients (48% female, 64% Non-Hispanic White, 17% Hispanic) with SS were identified. The incidence rate was 1.5-1.8/million/person-years and was stable over time, corresponding to an average of 530 new cases annually. The most common primary site was the extremity (*n* = 5877; 58%), and most patients presented with localized disease (*n* = 5753; 54%). The 5-year cause-specific survival was 60% across all races/ethnicities and 79% for localized, 57% for regional, and 12% for distant disease. Differences by race and ethnicity were found in the proportions of patients expected to be eligible for HLA-restricted cellular therapies targeting MAGE-A4. People of European/European descent had the highest estimated proportion (25%-39%), and people of Asian/Pacific Islander descent had the lowest (11%-17%).

**Conclusion:**

Engineered T-cells targeting MAGE-A4 have shown encouraging safety and efficacy in advanced SS; however, eligibility restrictions will lead to racial and ethnic disparities. HLA-independent solutions must be developed to counter disparities and ensure all patients have access.

**Implications for practice**

Synovial sarcoma is a rare subtype that predominantly affects adolescents and young adults (AYAs) with poor outcomes in advanced disease. Cellular therapies, such as engineered T-cells (TCRs) against MAGE-A4, have shown encouraging safety and efficacy; however, eligibility restricted based on HLA-A*02 type will compound existing racial and ethnic disparities in cancer care. TCRs that target additional HLA types or engage with a broader range of HLA-A*02 alleles are sorely needed. Continued development of HLA-independent cancer vaccines (including mRNA vaccines) and bispecific T-cell engagers may ensure all patients have access to promising therapies while proactively countering anticipated disparities.

## Introduction

Synovial sarcoma (SS) is a rare subtype of soft tissue sarcoma with a median age at diagnosis between 35 and 38 years.^1,2^ SS can arise anywhere in the body and most commonly presents in the extremities.^2^ The tumor is defined by the presence of a translocation t(X,18), which results in an aberrant SS18:SSX fusion protein that promotes tumorigenesis.^2^

SS is treated with a combination of local and systemic therapies, with some heterogeneity in management. For patients with localized disease, the 5-year overall survival (OS) has previously been estimated as 76%, compared with a 5-year OS of 10% for metastatic disease.^3^ Controlling advanced SS requires systemic therapy, typically with chemotherapies such as doxorubicin and ifosfamide, but most patients will ultimately progress.^2,3^ Of note, race and socioeconomic status have been associated with worse overall survival in some studies of synovial sarcoma patients.^4-7^

As of 2024, cellular therapies are newly approved for the treatment of advanced SS.^8^ T-cells with engineered T-cell receptors (TCRs) against highly-expressed cancer testis antigens, such as melanoma-associated antigen A4 (MAGE-A4)^9,10^ and New York esophageal squamous cell carcinoma 1 (NY-ESO-1),^11,12^ have shown safety and efficacy. Based on the results of the open-label Phase 2 study SPEARHEAD-1,^10^ the Food and Drug Administration (FDA) has granted accelerated approval to afamitresgene autoleucel (TECELRA, Adaptimmune, LLC), or afami-cel, for adults with advanced SS who have received prior chemotherapy and have specific human leukocyte antigen (HLA) allele (HLA-A*02:01, 02:02, 02:03, or 02:06), absence of a homozygous or heterozygous HLA-A*02:05 allele, and whose tumors express MAGE-A4 antigen.^13^

HLA-specific inclusion criteria are necessary for TCRs to allow for targeting of intracellular antigens, such as cancer testis antigens. Engineered TCRs are activated by interacting with peptides presented by the major histocompatibility complex (MHC) class I molecules on the tumor cell’s surface. These MHC class I molecules are categorized into HLA allelic families, the classical ones being HLA-A, HLA-B, and HLA-C, which are then further divided based on specific alleles.^14-16^

Initial TCRs have been developed to target the most common MHC class I alleles within allele families, in particular HLA-A*02,^17^ given its high prevalence in the global population.^14,16^ Because of this receptor specificity, a given TCR may only target a limited subset of a given allele family, such as HLA-A*02:01 and similar alleles within the HLA-A*02 family.^14,17^ Further, not all similar alleles may be included: for example, in SPEARHEAD-1, patients with the presence of an HLA-A*02:05 allele were excluded due to preclinical data demonstrating that TCRs targeting MAGE-A4 demonstrated alloreactivity toward multiple HLA-A*02:05-expressing cell types.^18^

While these HLA-A*02 subfamilies were selected due to global population prevalence, they are not evenly distributed in the population, and this may exacerbate pre-existing disparities in SS. For example, recent estimates show that the likelihood of patients being eligible for treatment or trial participation with HLA-A*02 restricted products is roughly one-half, but only one-third of African Americans are likely to be eligible.^19^ In addition to HLA-associated disparities, studies suggest some racially and ethnically minoritized people with cancer are less likely to meet multiple benchmarks for early diagnosis and trial access, such as: present with curative disease^20,21^; survive as long as non-minoritized cancer patients^20,21^; have the testing necessary to promote trial recruitment^22^; be knowledgeable of or interested in trials^23-25^; or be offered trial participation or enrolled in clinical trials.^26-30^ Therefore, HLA-associated disparities may compound already existing disparities in oncology clinical care and trial participation.

To define both baseline disparities in SS and identify the potential effect of disparate access to novel HLA-restricted therapies, we performed an analysis of the US Cancer Statistics (USCS) public access data registries to estimate the proportion of patients with advanced SS who are expected to be eligible for cellular therapy by race and ethnicity.

## Materials and methods

### Population and data

Aggregate data were obtained from the US Cancer Statistics (USCS) public access data registries^31^ of the Center for Disease Control and Prevention’s (CDC) National Program of Cancer Registries (NPCR)^32^ and National Cancer Institute’s (NCI) Surveillance, Epidemiology, and End Results Program (SEER).^33^ All cases from years 2001 to 2020 with the following ICD-O-3 codes for synovial sarcoma were included: NOS (9040/3), spindle cell (9041/3), epithelioid cell (9042/3), and biphasic (9043/3). For cases diagnosed from 2003 to 2017, 100% of the population is covered for all 50 U.S. states and the District of Columbia. In 2001 and 2002, cases that were diagnosed in Mississippi are not available, so 99% of the U.S. population is covered. Statistics based on fewer than 16 cases were suppressed to ensure privacy.

For a subset of cases, survival data were available within the SEER 17 (previously SEER 18) database, which includes data from 17 state or regional cancer registries.^34^ SEER 17 covers 26.5% of the US population and covers the following proportions of each race and ethnicity: White (22.4%), Black (23.1%), American Indian/Alaska Native (49%), Asian (70.7%), Native Hawaiian/Pacific Islander (70.3%), and Hispanic (66.3%).^35^ SEER 17 survival data was aggregated across all races/ethnicities. SEER-recommended race and origin coding was used to group patients based on racial/ethnic groups, including: (a) Non-Hispanic White (NHW), (b) Non-Hispanic Black (NHB), (c) Non-Hispanic American Indian/Alaska Native (NHAIAN), (d) Non-Hispanic Asian or Pacific Islander (NHAPI), and (e) Hispanic (all races). These categories were bridged to the geographic/ancestral/ethnic population groups in the Common, Intermediate, and Well-Documented HLA Alleles in World Populations (CIWD) Version 3.0.0 as follows: (a) NHW to “European/European descent,” (b) NHB to “African/African American,” (c) NHAIAN to “Native American,” (d) NHAPI to “Asian/Pacific Islands,” and (e) Hispanic (all races) to “South or Central America/Hispanic/Latino.”

### Statistical analysis

For incidence rate calculations, data were age-adjusted to a year 2000 US standard population (19 age groups—Census P25-1130). Confidence interval (CI) estimations were performed using the Tiwari method.^36^ Percentage changes were calculated using 1-year averages for each endpoint; annual percentage change (APC) was calculated using the least squares method. The total number of patients diagnosed with synovial sarcoma from 2001 to 2020 was obtained, as was the number and proportion of patients based on age, race, primary site, and extent of disease (local, regional, distant).

Survival analyses were performed using SEER 17 data with actuarial methods at 1, 2, 3, 4, and 5 years post-diagnosis with 95% CI. Survival rates were calculated across all races/ethnicities by extent of disease. Rates for survival and extent of disease were also calculated by race/ethnicity (NHW, NHB, NHAIAN, NHAPI, Hispanic).

The estimated proportion eligible for cellular therapy was calculated using previously published estimates from the CIWD Version 3.0.0, which is a central registry compiled from over 8 million people across 20 hematopoietic stem cell donor registries.^16^ The Hardy Weinberg principle was used to estimate the frequency by race and ethnicity of genotypes with at least one allele of HLA-A*02:01, 02:02, 02:03, or 02:06.^37^ These frequencies were combined to give a total estimate of eligible allele frequency. Finally, we used the minimum and maximum of a range of previous estimates of MAGE-A4 positivity in patients with SS—53% to 82%—to adjust these estimates by antigen prevalence.^38-40^ The estimated proportion of patients expected to be eligible for cellular therapy based on HLA type and MAGE-A4 positivity was then calculated in total and by race and ethnicity.

### Software

SEER*Stat software version 8.4.3 was used to extract data released on November 8, 2023. Statistical analyses were performed within SEER*Stat.^41^ Graphs were generated using GraphPad Prism version 10.1.1 (Dotmatics).

## Results

### Incidence, characteristics, and survival prior to cellular therapy era

**Figure 1.**
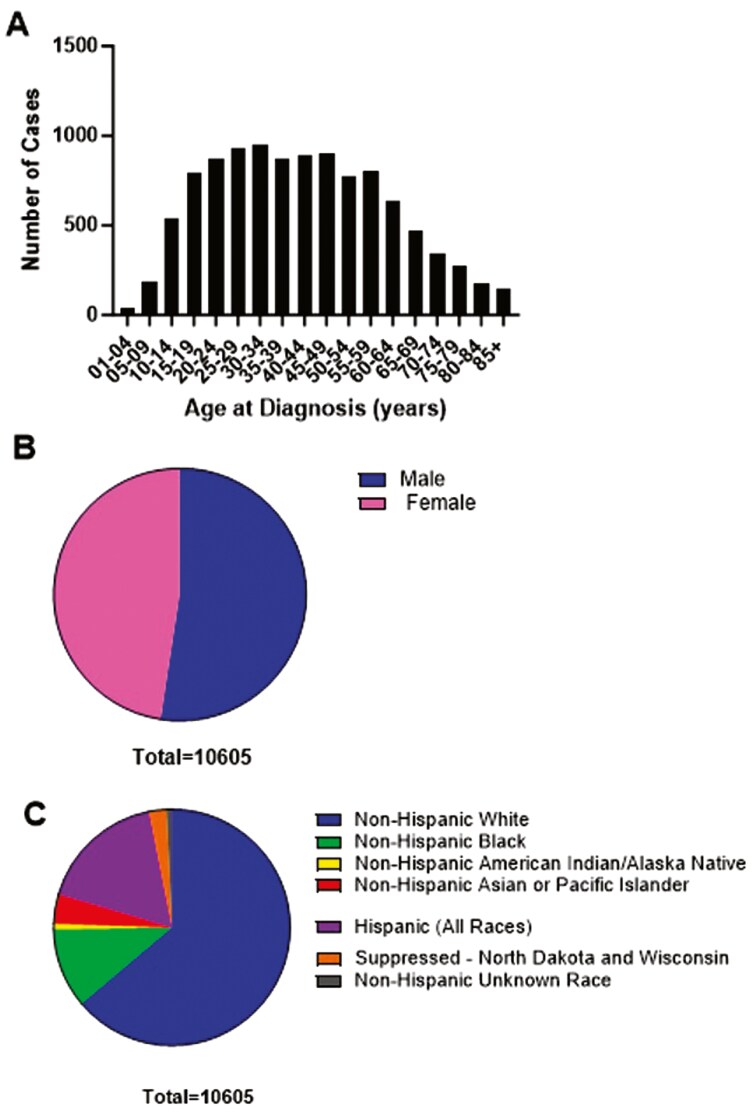
Incidence of synovial sarcoma by (a) age (b) gender and (c) race and ethnicity.

From 2001 to 2020, 10 605 patients (48% female) were diagnosed with SS. Of these total patients, 6,769 (64%) were non-Hispanic White, 1158 (11%) were non-Hispanic Black, 427 (4%) were non-Hispanic Asian or Pacific Islander, 82 (1%) were non-Hispanic American Indian or Alaska Native, 1838 (17%) were Hispanic (all races), 80 (1%) were non-Hispanic but of unknown race, and 251 (2%) were of unknown race and ethnicity. SS occurred across all ages, with the mode of cases between ages 30-34 (953 or 9%) and 25-29 (927 or 9%) (**Figure 1**).

The range of annual new cases ranged from 417 (in 2001) to 601 (in 2014), with a median new cases of 527 per year. The annual incidence rate ranged from 1.5to 1.8 per 1 000 000 people during this period, with an annual percentage change of 0% (95% CI −0.5% to 0.5%) (Supplementary Figure S1).

The most common primary site was soft tissue of the extremities (*n* = 5,877; 58%), followed by soft tissue of the thorax/abdomen/pelvis/other (*n* = 2404; 24%), head & neck (*n* = 534; 5%), lung and pleura (*n* = 735; 7%), and kidney/retroperitoneum (*n* = 186; 2%). Fifty-four percent of patients (*n* = 5753) presented with localized disease, 21% (*n* = 2,278) with regionally advanced disease, 17% (*n* = 1789) with distant disease, and 7% with unknown disease extent (*n* = 782) (Supplementary Figure S2).

There was no statistically significant difference in the proportion of patients with localized, regional, or advanced disease at presentation by race or ethnicity. The proportion with distant disease was numerically higher for Non-Hispanic Black (21%) compared to Non-Hispanic White (17%). The proportion with distant disease was 12% for Non-Hispanic Asian or Pacific Islander, 16% for Hispanic patients (all races), and could not be estimated for Non-Hispanic American Indian/Alaska Native patients, as there were fewer than 16 cases and thus suppressed by SEER.

Survival information was available in SEER for 2601 cases (25% of the data available for incidence calculations), which included 1250 localized (48%), 521 regional (20%), and 381 distant (15%) cases. Of these, 91 (3%) cases were unstaged and 358 (14%) were blank.

**Figure 2.**
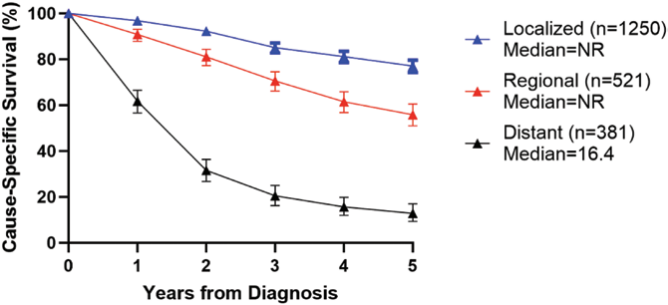
Cause-specific survival by extent of disease (localized, regional, distant) at 1, 2, 3, 4, and 5 years after diagnosis.

**Figure 3.**
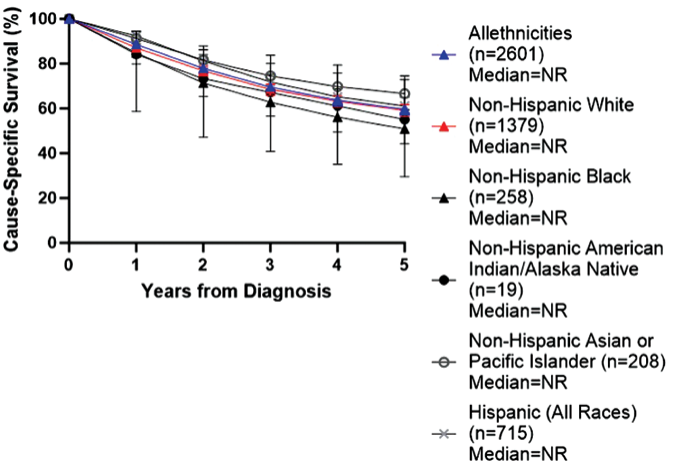
Cause-specific survival by race and ethnicity at 1, 2, 3, 4, and 5 years after diagnosis.

5-year survival was 60% across all patients, with significant differences by disease extent at presentation: 76% for localized, 54% for regional, and 13% for distant disease, respectively (Figure 2). 5-year survival was not statistically significant across race and ethnic groups, but the 5-year survival was numerically higher for Non-Hispanic Whites (59%) and Non-Hispanic Asian or Pacific Islanders (67%) compared to Non-Hispanic Blacks (51%) and Non-Hispanic American Indian/Alaska Natives (55%). The 5-year-survival for Hispanics (all races) was 61% (**Figure 3**).

### Eligibility for HLA-restricted cellular therapies

According to the Common, Intermediate, and Well-Documented HLA Alleles in World Populations (CIWD) Version 3.0.0 and the Hardy-Weinberg principle, the proportion of people with at least one HLA-A*02:01, 02:02, 02:03, or 02:06 allele by CIWD combined geographic, ancestral, and ethnic groups was 47.3% for European/European descent; 29.5% for African/African American descent; 40.9% for Native American descent; 20.7% for Asian/Pacific Islands descent; 42.1% for South or Central American, Hispanic, or Latino descent; and 33.4% for Middle East/North Coast of African descent.^16,37^

The individual allele frequencies of HLA-A*02:01,:02,:03,:06, and:05 by population group are demonstrated in **Supplementary Table S1**. It was not feasible to calculate and subtract the compound heterozygosity of the eligible alleles with the exclusionary allele HLA-A*02:05 due to limitations of reporting in the CIWD, though the proportion of patients expected to have HLA-A*02:05 was low across all races and ethnicities.

The estimated proportion of patients who are expected to be eligible for cellular therapy based on HLA-A*02 allele and a range of MAGE-A4 positivity estimates (53%-82% of patients with SS) is summarized in Table 1. Differences by race were found in the proportions of patients expected to be eligible. The European/European descent population group had the highest estimated proportion (25.1-38.8%), while the Asian/Pacific Islands group had the lowest estimated proportion (11.0-17.0%).

**Table 1.** Estimated proportion of patients eligible for HLA-A*02 allele-restricted MAGE-A4 targeting TCR-T cellular therapy by CIWD combined geographic/ancestral/ethnic population groups.

CIWD population group	Lower estimate (%)	Higher estimate (%)
All groups	22.9	35.4
European/European descent	25.1	38.8
South or Central America/Hispanic/Latino	22.3	34.5
Native American populations	21.7	33.5
African/African American	15.6	24.2
Asian/Pacific Islands	11.0	17.0

## Discussion

In this US population-based analysis, we find that HLA-based restrictions have a differential impact on eligibility for TCR cellular therapy by race and ethnic origin groups. Additionally, we confirm prior work describing synovial sarcoma as a rare sarcoma subtype predominantly affecting young adults and highlight poor prognosis for patients with synovial sarcoma, particularly those with advanced or metastatic disease.^1-3^ Though not statistically significant, we found a numerically higher rate of presentation with distant disease in Non-Hispanic Black patients compared to Non-Hispanic White patients, which may then be compounded by disparities in eligibility for HLA-restricted therapies.

Our findings highlight an urgent need for novel therapies, including cellular therapies, for advanced SS. Based on the results of an open-label phase 2 trial,^10^ the FDA recently granted accelerated approval to afami-cel, and it is now commercially available for clinical use at specialized centers across the United States.

Despite demonstrated efficacy, these therapies are restricted to patients with specific HLA-A*02 alleles. Prior studies have shown that HLA-restriction therapies are prone to racial and ethnic disparities.^19,42^ Our study supports this in advanced SS. Across all races and ethnicities, only 22.9%-35.4% of patients in need of salvage therapy would be expected to be eligible for an HLA-A*02 restricted product targeting MAGE-A4. Non-Hispanic White patients would be expected to have the highest proportion eligible; Non-Hispanic Asian or Pacific Islander would be expected to have the lowest proportion eligible. This estimate confirms the finding that only 28% of patients pre-screened for the SPEARHEAD-1 had an eligible HLA-A*02 allele for therapy and deepens understanding of which populations are most affected. Disparities in HLA presentation by racial and ethnic status will likely further compound existing disparities in clinical presentation and outcome,^20,21^ as well as clinical trial recruitment and enrollment.^26-30^

Given the approval for these engineered TCRs in advanced SS, it is important to consider how to proactively counter these anticipated disparities, in particular for Non-Hispanic Asian or Pacific Islander populations, in which only 11%-17% of patients are expected to be eligible. One option may be to encourage cellular therapy companies to develop TCRs effective across other HLA subtypes. A limitation to this option is the significant cost associated with developing and testing different products. Manufacturing of engineered TCRs is highly complex, regulated, and costly, and it can take 3 or more weeks to produce TCRs for clinical use in each patient. Thus, it makes financial and operational sense that commercial products have focused primarily on the most common HLA-A*02 alleles.^17^ However, testing compatibility with a broader range of alleles within the HLA-A*02 family or targeting a broader range of HLA-A alleles may be strategies to expand access. Additionally, focusing development on HLA-A alleles that are common in parts of the world where HLA-A*02 is less common may be a more efficient way to expand access; however, these efforts would require concomitant efforts toward addressing the resource constraints to administering TCRs in a global setting. Examples of these are HLA-A*24:02, which is very frequent in all regions but Sub-Saharan Africa, including areas of Africa and Asia where HLA-A*02:01 is less common, and HLA-A*30:02, which is very frequent in Sub-Saharan Africa.^15^

Alternate targeted therapies for SS may be one strategy to mitigate this impact, but they have some limitations. TCRs to other antigens such as NY-ESO-1 and PRAME relevant to sarcomas are also HLA-restricted in development to date.^43,44^ Non-TCR strategies have generated some interest as a possible means of targeting MAGE-A4 or other highly expressed antigens in an HLA-independent manner, including CMB305, a prime-boost vaccine regimen against NY-ESO-1,^45,46^ and bispecific T-cell engagers (BiTEs) such as pAXLxCD3ε, which targets the AXL tyrosine kinase receptor highly expressed on many sarcoma subtypes.^47^ Personalized mRNA vaccines have also been of broad interest in this space.^48^ Limitations of these strategies are their relative novelty and limited clinical data to support their use to date, as well as the potential for lesser durability or efficacy relative to a cell therapy product.

Our study has some limitations. The analysis was conducted based on aggregate data from a national population-based sample of cancer patients, and the small sample size in the survival population likely results in inadequate power to detect survival differences based on race and ethnicity. In addition, the proportion of data entries from specific races and ethnic groups in the SEER 17 database may not represent the overall proportion of all patients with synovial sarcoma in the US. Synovial sarcoma is a rare subtype of sarcoma, so alternative ICD-10 coding (ie, “C49: malignant neoplasm of connective and soft tissue”) is possible, and these patients would not be included in our analysis. The separate reporting of each HLA-A*02 allele without additional information on coinheritance means that our estimate may overestimate inclusion based on the number of patients who may be compound heterozygotes for multiple included alleles. Similarly, we were not able to include compound heterozygosity with the exclusionary allele HLA-A*02:05, which may underestimate HLA-based exclusions given the greater prevalence of this allele in people of African/African American and Middle Eastern/North African descent. Given the relatively lower prevalence of these alleles, we would anticipate this to have a smaller effect on these estimates. We relied on published estimates of HLA-A*02 serotypes by reported ancestry, but notably, the published estimates of origin and ancestry used in the CIWD are not precisely matched to SEER classification of race and ethnicity and may not perfectly reflect allelic frequency in the US population. Additionally, because patients of Middle East/North African nationalities are included in the SEER Program Coding and Staging Manual 2023 under the Non-Hispanic White category, population-level data on the prevalence of SS is not disaggregated for this population. This aggregation prevents our study from including the effect of lower eligibility in patients of Middle Eastern/North African ancestry in the SEER “Non-Hispanic White” category. Finally, MAGE-A4 positivity is a requirement for treatment eligibility, and further research is needed on which cutoffs best predict clinical response.

## Conclusion

Synovial sarcoma is a rare sarcoma subtype that predominantly affects adolescents and young adults (AYAs). Survival rates for advanced SS are exceedingly low, and nearly all patients will ultimately progress on conventional chemotherapy. Novel cellular therapies, such as engineered T-cells against MAGE-A4, have shown encouraging safety and efficacy in advanced SS. Unfortunately, eligibility restricted based on HLA-A*02 type will likely compound existing racial and ethnic disparities in clinical trial recruitment and enrollment. TCRs that target additional HLA types or engage with a broader range of HLA-A*02 alleles are sorely needed, alongside interventions to promote clinical trial recruitment, enrollment, and retention for underrepresented patient populations.^49^ Continued development of HLA-independent cancer vaccines (including mRNA vaccines) and bispecific T-cell engagers may ensure all patients have access to these promising therapies while proactively countering these anticipated disparities.

## Supplementary Material

oyaf193_suppl_Supplementary_Figures_1

oyaf193_suppl_Supplementary_Figures_2

oyaf193_suppl_Supplementary_Tables_1

## Data Availability

The data underlying this article will be shared on reasonable request to the corresponding author.
