# Supplementary material for: Effect of HLA restriction on racial and ethnic disparities in access to immune therapies for advanced synovial sarcoma
Source: Oncologist. 2025 Jul 16;30(7):oyaf193. doi: 10.1093/oncolo/oyaf193 (PMC12265472; doi:10.1093/oncolo/oyaf193)
Supplement: oyaf193_suppl_Supplementary_Figures_1 [file oyaf193_suppl_supplementary_figures_1.docx]

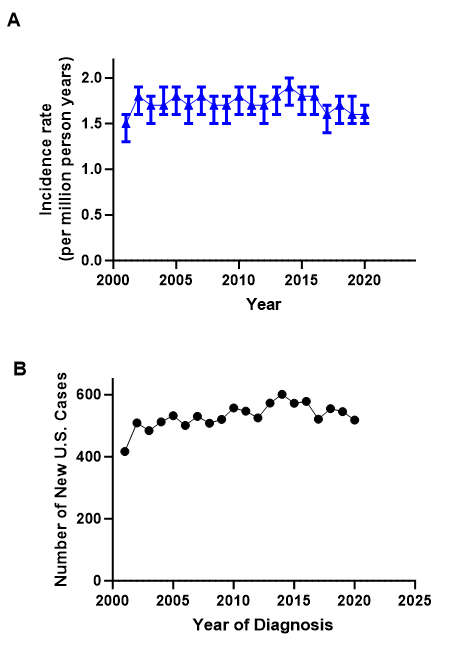


Supplemental Figure 1. (a) Annual incidence rate of synovial sarcoma and (b) newly diagnosed cases per year from 2001-2020
