# Supplementary material for: Effect of HLA restriction on racial and ethnic disparities in access to immune therapies for advanced synovial sarcoma
Source: Oncologist. 2025 Jul 16;30(7):oyaf193. doi: 10.1093/oncolo/oyaf193 (PMC12265472; doi:10.1093/oncolo/oyaf193)
Supplement: oyaf193_suppl_Supplementary_Figures_2 [file oyaf193_suppl_supplementary_figures_2.docx]

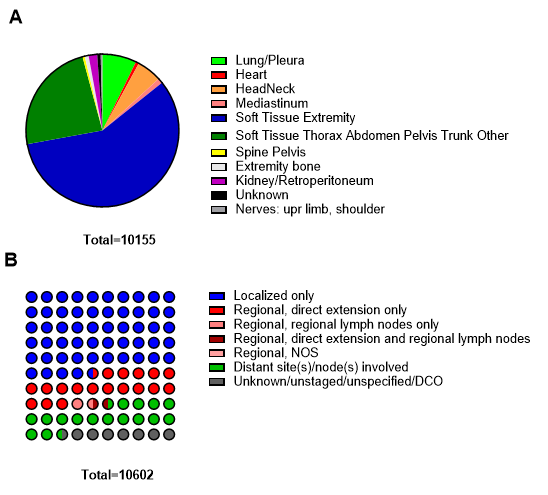


Supplemental Figure 2. (a) Location of primary site of synovial sarcoma and (b) extent of disease at time of diagnosis
