# Supplementary material for: Effect of HLA restriction on racial and ethnic disparities in access to immune therapies for advanced synovial sarcoma
Source: Oncologist. 2025 Jul 16;30(7):oyaf193. doi: 10.1093/oncolo/oyaf193 (PMC12265472; doi:10.1093/oncolo/oyaf193)
Supplement: oyaf193_suppl_Supplementary_Tables_1 [file oyaf193_suppl_supplementary_tables_1.docx]

Supplemental Table 1. Allele Frequency by CIWD Population Group.

| **Allele** | **AFA** | **API** | **EURO** | **MENA** | **HIS** | **NAM** | **UNK** | **Total** |
| --- | --- | --- | --- | --- | --- | --- | --- | --- |
| **A*02:01** | **0.118810** | **0.065069** | **0.271100** | **0.173514** | **0.198648** | **0.194368** | **0.218159** | **0.240648** |
| **A*02:02** | **0.040214** | **0.000249** | **0.000774** | **0.005449** | **0.006187** | **0.010228** | **0.004597** | **0.002389** |
| **A*02:03** | **0.000201** | **0.019938** | **0.000042** | **0.000296** | **0.000114** | **0.000672** | **0.001145** | **0.001744** |
| **A*02:06** | **0.001274** | **0.024385** | **0.001940** | **0.004552** | **0.033904** | **0.025668** | **0.006910** | **0.005687** |
|  |  |  |  |  |  |  |  |  |
| **A*02:05** | **0.016248** | **0.005378** | **0.007522** | **0.025385** | **0.013184** | **0.013752** | **0.013568** | **0.008775** |

Population groups: AFA (African/African American), API (Asian/Pacific Islands), EURO (European/European descent), MENA (Middle East/North Coast of Africa), HIS (South or Central America/Hispanic/Latino), NAM (Native American populations) and UNK (unknown/not asked/multiple ancestries/other). Total is the overall population i.e., all groups combined.
